# Lower Extremity Rehabilitation in Patients with Post-Stroke Sequelae through Virtual Reality Associated with Mirror Therapy

**DOI:** 10.3390/ijerph18052654

**Published:** 2021-03-06

**Authors:** Roxana Steliana Miclaus, Nadinne Roman, Ramona Henter, Silviu Caloian

**Affiliations:** 1 Faculty of Medicine, Transilvania University of Brașov, 500036 Brașov, Romania; roxicum@unitbv.ro (R.S.M.); caloian.silviu@unitbv.ro (S.C.); 2 Faculty of Psychology and Education Sciences, Transilvania University of Brașov, Str. N. Bălcescu 56, 500368 Brașov, Romania; ramona.henter@unitbv.ro

**Keywords:** rehabilitation, post-stroke, virtual reality, mirror therapy, lower extremity

## Abstract

More innovative technologies are used worldwide in patient’s rehabilitation after stroke, as it represents a significant cause of disability. The majority of the studies use a single type of therapy in therapeutic protocols. We aimed to identify if the association of virtual reality (VR) therapy and mirror therapy (MT) exercises have better outcomes in lower extremity rehabilitation in post-stroke patients compared to standard physiotherapy. Fifty-nine inpatients from 76 initially identified were included in the research. One experimental group (*n* = 31) received VR therapy and MT, while the control group (*n* = 28) received standard physiotherapy. Each group performed seventy minutes of therapy per day for ten days. Statistical analysis was performed with nonparametric tests. Wilcoxon Signed-Rank test showed that both groups registered significant differences between pre-and post-therapy clinical status for the range of motion and muscle strength (*p* < 0.001 and Cohen’s d between 0.324 and 0.645). Motor Fugl Meyer Lower Extremity Assessment also suggested significant differences pre-and post-therapy for both groups (*p* < 0.05 and Cohen’s d 0.254 for the control group and 0.685 for the experimental group). Mann-Whitney results suggested that VR and MT as a therapeutic intervention have better outcomes than standard physiotherapy in range of motion (*p* < 0.05, Cohen’s d 0.693), muscle strength (*p* < 0.05, Cohen’s d 0.924), lower extremity functionality (*p* < 0.05, Cohen’s d 0.984) and postural balance (*p* < 0.05, Cohen’s d 0.936). Our research suggests that VR therapy associated with MT may successfully substitute classic physiotherapy in lower extremity rehabilitation after stroke.

## 1. Introduction

Stroke is a significant cause of disability, with millions of stroke patients burdened with permanent neurological deficits, mainly motor and psychological. In most cases, post-stroke recovery requires long-time interventions in a multidisciplinary team, primarily depending on the severity of the stroke, the associated pathologies, the patient’s age, the time since stroke and the beginning of rehabilitation [1,2]. Neurorehabilitation after a stroke engages the recovery of the motor deficit and the recovery of language function, cognitive recovery, sensory and sphincter functions, and the functional reintegration as much as possible, as active as possible, into family and socio-professional life components. Post-stroke rehabilitation in the subacute phase begins when the patients’ are clinically balanced and stable, especially with regards to cardiorespiratory functions, and last but not least, when the tasks can be understood and supported by the patients’ participation and involvement in the rehabilitation program. It starts with the fifth week after stroke and has a relative duration of about three months [3]. Also, significant features in the physical rehabilitation of post-stroke patients are related to behavioral, cognitive and contextual factors that should be taken into account when planning therapy [4,5].

Rehabilitation in the chronic phase begins after the patient has passed the subacute stage entering the chronic phase, followed by a constant and consistent rehabilitation program necessary to be continued throughout his life. The maximum level of complexity of the physiotherapy program is reached in the chronic phase when the highest acquisition and velocity of motor improvement are also expected. Regardless of the pathology stage, the basic principle is to stimulate and activate somatic structures through different activities and tasks. The most commonly used post-stroke rehabilitation techniques usually refer to correct posture, avoiding synkinesis, increasing muscle strength, active mobilization, proprioception, balance, and daily activities training. All these techniques and objectives are achieved in rehabilitation programs designed to repeatedly challenge the body during the day and through relaxation and a correct posture during the night [6,7].

Mirror therapy (MT) is a type of rehabilitation method which activates the so-called mirror neurons. By visualizing in the mirror, the movement of the opposite healthy limb, the mirror neurons receive the necessary feedback to initiate the process of neuroplasticity. The healthy extremity is placed in front of a mirror and the illusion of the movement of the paretic side is created when the healthy part is activated. MT showed good results on motor deficiencies, as well as on sensations, visual-spatial neglect and pain after stroke [8].

Therefore, MT is a method successfully used in the post-stroke patient’s rehabilitation. This method, along with virtual reality (VR), is used to improve the motor function of the lower extremity (LE), helping to regain balance, stability, and coordinated gait by training the muscles responsible for these activities. MT has been shown to improve the voluntary control of the impaired lower limb, especially the ankle joint by amplifying information (visual pathway, motor pathway, proprioceptive pathway), which determine a more complex efficiency of neurological deficit recovery. By applying MT to the lower limbs affected by stroke, it was observed the improvement of the stability of the patients, as the lower limbs play an essential role in performing ambulation and stability during daily activities. This approach exploits the brain’s preferences to prioritize the visual reaction over the somatosensory reaction regarding limb position [9,10,11]. New research is evaluating MT efficiency when combined with other techniques, such as cognitive therapeutic exercise, and other research shows that MT combined with other types of therapies has a better effect on the physical rehabilitation of post-stroke patients [12,13].

VR is a new technology involving several scientific fields’ collaboration: biomechanics, internet technology engineering, rehabilitation and cognitive neuroscience. One of the most applicable areas of this modern technology is the medical field. VR has emerged as a new treatment approached in stroke rehabilitation, assuming the use of exercise programs designed to simulate real-life objects and activities using a computer [14]. This new approach is very advantageous as recovery programs design provided by the new environment and seems to be more exciting and enjoyable than traditional physiotherapy tasks, thus encouraging more repetition and involving the patient in the therapeutic program through “gamification” [15,16]. Patients become more motivated as they achieve increasingly performances and complexity.

VR is a technology that allows the user to interact with a computer-simulated environment, whether that environment is a simulation of the real or merely an imaginary world, which influence the patient’s visual and proprioception feedback mechanism, therefore, facilitating the therapy outcomes. The VR therapy involves a computer generation of a virtual environment capable to interact with the patients through the sensory-motor functions. Real-time interaction with a multidimensional and multisensory environment is the critical element of VR therapy. The patient interacts not only with the virtual environment but also with various objects that are part of that environment [17,18].

VR began to be successfully used in post-stroke patient’s rehabilitation due to the many advantages it entails:

(a) the access in a safe environment to real-life situations, otherwise inaccessible to patients due to cognitive, motor and psychological limitations
(b) the possibility to alter the exercises, to emphasize specific movements during patient execution, thus becoming more comfortable to perform,
(c) the unique and personalized character of the exercises from one patient to another [19].

VR technology is currently being explored for its potential benefits as a therapeutic intervention for training coordinated movement patterns, as well as for its entrepreneurial outcomes [20]. This technology offers the ability to create an environment in which the intensity of feedback and training can be systematically manipulated and improved to create the most appropriate paradigm of individualized motor learning. Most literature reviews have shown that VR has good results in post-stroke patients’ therapy, especially as an adjunct to classical physiotherapy [21,22,23].

Researches address the burden of the individuals and families of the society and the health care systems concerning post-stroke patients who have disabilities and often can no longer reintegrate into socio-professional activities. Therefore, more and more emphasis is placed on developing facilities to speed patients’ physical rehabilitation after stroke [24,25,26]. Therefore, this study aims to determine the effectiveness and particularities of the use of VR associated with MT in the recovery of the LE of patients with post-stroke sequelae, compared to a standard physiotherapy program, as a novel approach in LE post-stroke rehabilitation, to contribute to the amendment of the efficient techniques and methods used in post-stroke rehabilitation.

## 2. Materials and Methods

### 2.1. Study Design

The study was prospective, randomized trial, conducted over nine months from July 2019 to March 2020 on the Neurorehabilitation Clinical Department of the Clinical Hospital of Psychiatry and Neurology in Brasov, Brasov County, Romania. The patients were introduced in the therapy one by one (asynchronously) as soon as they reach the inclusion criteria. The Research and Ethics Committee of the Clinical Hospital of Psychiatry and Neurology in Brasov approved the study (no. 12534/18 July 2019). Each patient signed the Informed consent after they acknowledged his/her valuable participation in increasing the quality of life of future patients. Also, patients were informed in advance regarding the possibility of drop out from the study at any moment. The study was registered in clinicaltrials.gov, with no. NCT04436770.

### 2.2. Participants

Initially, seventy-six patients were selected for the study, out of whom, twelve were excluded based on the exclusion criteria; sixty-four patients were admitted into the study. The inclusion criteria were:

(1) stroke survivors after the subacute phase, at least six months post-stroke [27], and less than four years. This time frame is the best choice for functional rehabilitation and patients inside this frame time have the best status for rehabilitation, especially VR (stroke survivors with poor rehabilitation have spasticity, stiffness, tissue retraction, joint misalignments over more than four years of stroke that prevent the application of new techniques as VR and MT)
(2) assessment criteria: at least 20-degree hip flexion and 10 degrees hip abduction against gravity, and at least 30-degree knee flexion against gravity.

The exclusion criteria were: severe cognitive impairments, global or transcortical sensory aphasia, anaemia, atrial fibrillation, imbalance of anticoagulant treatment, epilepsy, NYHA class IV heart failure, other dysfunctions in the lower extremity such as surgery, fractures, severe osteoarthritis, periarthritis or moderate-severe pain.

Sixty-four participants were assigned to the experimental and control groups, using simple randomization. To avoid bias within our sample (*n* = 64), we used GraphPad QuickCalcs to generate numbers that assigned patients into the two groups. The allocation was performed using sealed opaque envelopes with the group name, which were placed in a plastic container in numerical order. The randomization procedure was performed by different individuals who were not involved in the work [28]. During the research, five participants were identified as having different health conditions not allowing them to continue participating in the study. Two patients manifested anemia and atrial fibrillation, two patients developed imbalance of anticoagulant medication and one manifested epileptic crisis. Therefore, the rest of fifty-nine patients took part in the entire research program. The participants were divided into two groups: Experimental group (*n* = 31) and Control group (*n* = 28).

**Figure 1:**
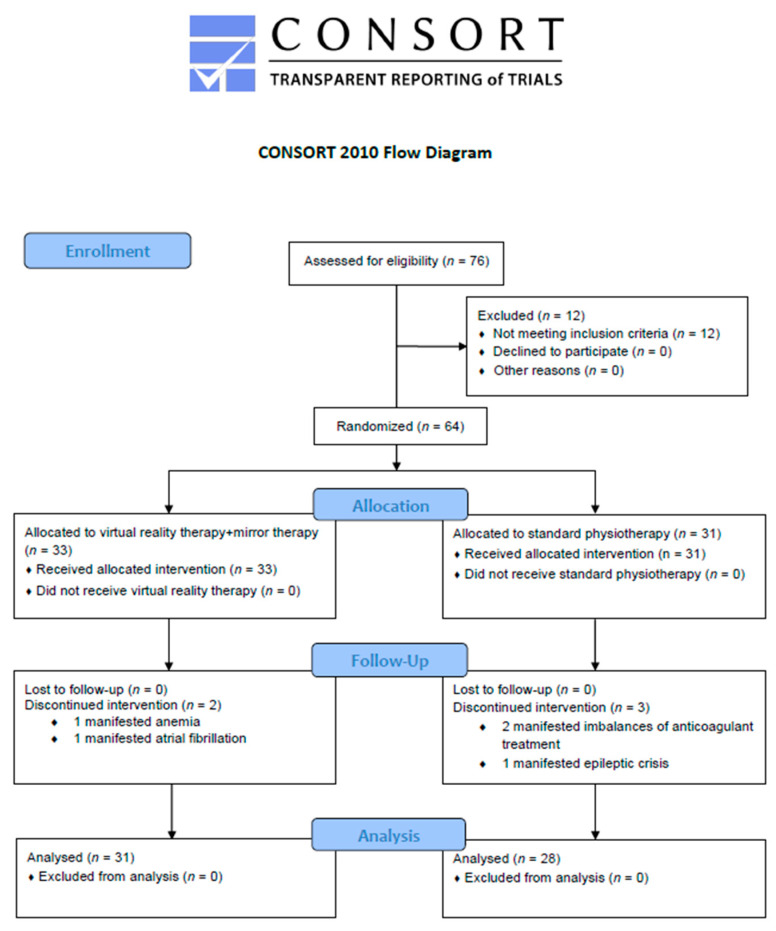
the CONSORT flow diagram.

All data regarding groups allocation is presented in Figure 1, the CONSORT flow diagram. The duration of participation in the study for every patient was of 10 working days for two consecutive weeks. Each group received a 70-min therapy session for the LE for ten days. Both groups received 20 min of ergometer bicycle and treadmill training. The control group benefited daily from a standard physiotherapy protocol of exercises such as self-passive and assistive mobilization, active mobilization, and active mobilization with resistance for LE, proprioception, motion control, and coordination for a total time of 70 min. The additional program of the experimental group included 27 to 37 min of VR therapy (this duration was set according to the assessment of patient’s capacity) associated with MT exercises (13 to 23 min), so overall every patient performed seventy minutes of daily LE training. The protocol for MT therapy was set to analytical motion with the healthy ankle, but also exercises of proprioception, motion control and coordination of LE. The types of interventions for each group are detailed in Table 1.

### 2.3. Outcome Measures

The research methodology consisted of the assessment undertaken with four psychometric scales: Functional Independence Measure (FIM), Modified Rankin Scale (MRS), Modified Ashworth Scale (MAS), and Fugl Meyer Lower Extremity Assessment (FMLE). Manual Muscle Testing (MMT) and Active Range of Motion (AROM) were used to assess muscle strength and range of motion. The assessments were carried out by two experienced physiotherapists specifically trained for this research and aimed to register data in scales on stroke severity, activities of daily living, degree of spasticity, motor function and functionality, and the active range of motion.

All assessment scales were found excellently related to other types of assessment, regarding the psychometric properties. Therefore, MRS was used to assess stroke severity (disability and dependence), FIM was used for the assessment of ADLs, MAS was used to assess the degree of spasticity, and LE motor function and functionality was assessed through FMLE [29,30,31,32]. Also, MMT reliability and validity proved to be a useful instrument to assess muscle motor force [33]. The Functional Reach Test (FRT) was used to measure reaching distance and is a simple tool providing information regarding balance capacity [34]. Time Up to Go (TUG) test was used to assess the fall risk and the progress of balance, siting, standing and walking. TUG test proved to have good intratester reliability and good construct validity in correlation with gait assessment [35].

### 2.4. Procedures

#### 2.4.1. Virtual Reality Software, Device and Exergames

The technology used consisted of a 55-inch TV screen, a computer running MIRA (a software for virtual reality therapy), and a Kinect sensor (Microsoft Corporation, Redmond, WA, USA,) which allows the detection of the human body, joints and movements on all three axes. MIRA (MIRA Rehab Limited., London, UK) is a VR rehabilitation tool, using the Kinect sensor to calibrate the patient’s position at the beginning of each VR session, or during exergames, if necessary. The technology used includes an evaluation tool for the AROM assessment, also performed through the Kinect sensor [36]. During the research, the MIRA software was updated from version 2.2.3.0 (released on 16 July 2019) to version 2.2.5.8 (released on 19 December 2019).

As described in recent research regarding upper extremity in post-stroke rehabilitation using MIRA [37], a physiotherapist using the technology assessed the patient’s AROM additionally to the other assessments, to establish the protocol of exergames used in VR therapy. Therefore, VR therapy use required the following steps:

(a) Assess AROM with a goniometer, assess MAS, MRS, FIM, FMLE and FRT
(b) Assess AROM through Kinect sensor and MIRA software
(c) Establish the limits of ROM performed during exergames based on the AROM assessment
(d) Establish the level of technology tolerance to motion pattern and correctness (the adjustment of the tolerance levels for motion from 0 to 100%. The lower the tolerance, the higher the software’s feedback on the correctness of movement, warning the patient that he/she is not performing the task accurately). The lower level of tolerance used in our research was set to 20%, according to the manufacturer’s recommendations, excepting the first two VR sessions, when the tolerance level was set to 50% to encourage the patient and to accustom him with the technology.
(e) Assigning the patient’s protocol of exergames by the results obtained from the assessments

The types of exergames used in the research were set based on three levels of difficulty:

(i) The most comfortable types of exergames, for patients with only 20 degrees of hip flexion, MMT 2–3, 10 degrees of hip abduction and 30 degrees of knee flexion.
(ii) The medium types of exercises, for patients with 25–60 degrees of hip flexion, 10–20 degrees of hip abduction, MMT 3, and 30–60 degrees of knee flexion.
(iii) The intense level of exergames, for patients with 60–90 degrees of hip flexion, and 20–30 degrees of hip abduction, MMT-4, and 60–90 degrees of knee flexion.

The games used were customized according to the functional capacity of patients, divided into three groups according to their AROM and MMT capacity: limited, low and high. For patients assigned to the easy set of exergames, we adjusted the virtual reality exergames during each session.

For the easy set of exergames a chair was used for the patient support on the healthy side, while performing the exercises with the affected LE. Furthermore, gradually adjustments for ROM were performed after 3–4 sessions of VR therapy for the hip and knee motions, according to the first values determined at the first assessment. The analytical pattern of motions for LE was used within the exergames.

For the medium types of exergames, a chair was used during half of the sessions, for the patient support on the healthy side, while performing the exercises with the affected LE, and the exergames were adjusted so that the subjects could do, as recreational activities, analytical movements of flexion, extension, abduction and hip rotation as well as analytical knee movements, against gravity.

Regarding the third level of types of exergames, exercises involving complex movements were adapted. The multiple movements of the hip joint, involving the entire lower limb, were performed in the frontal-anterior, sagittal and transverse plane. The elaborate motions were also adjusted to increase control and coordination so that movements could be performed diagonally, vertically and randomly.

For patients with MMT of at least 3, we increased the exergames difficulty level by attaching weights of 1–2 kg to the ankle, according to the patient’s strength. Throughout the virtual reality therapy sessions, the patients received specialized supervision and were initially guided verbally by a physiotherapist until they learnt how to perform the exergames correctly.

#### 2.4.2. Mirror Therapy Exercises

MT is performed by setting the paretic extremity behind a mirror which is positioned so that the healthy side’s reflection becomes visible in the place of the affected limb, which is continuously covered.

In the case of bilateral movement, additional stimulation of the affected unilateral cerebral cortex is induced. The use of the healthy side and the paretic extremity is determining an essential skill in restoring the lower limbs’ function the balance of proprioceptive impulses from simultaneous arousal.

For the first level of the exercises, analytical and coordination motions were performed for the ankle joint, with the healthy side. The exercises included dorsiflexion, plantar flexion, inversion, eversion.

The exercises for coordination and muscle control included a different pattern of motions performed at physiotherapists’ indication. Using a plate with five different coloured marks, placed diagonally, the patient touches alternately with the foot, once with the heel and once with the tip, the colours indicated by the therapist. We used a board with different drown animals, and the patient was instructed verbally to touch the board with the heel (dorsiflexion) or with the inner edge (eversion) on the indicated animal in random order. With the help of a balance board, the patient performs anteroposterior and lateral movements, emphasising dorsiflexion and eversion.

The second level of MT therapy was set for the patients with MMT < 3; all the exercises from level one was executed, performing the same motions with the affected limb (bilaterally).

The third level of MT was assigned for patients with MMT > 3; after performing the exercises from the first level, executed only with the healthy side, a weight of 0.500 g was added on the affected forefoot, and the same exercises were performed freely with the affected side, and also bilaterally. Examples of VR and MT therapy are displayed in the supplementary file.

### 2.5. Statistical Analysis

Firstly, we investigated the assumption of normal data distribution, using Shapiro-Wilk normality tests, and found that they were violated, so the statistical analysis involved nonparametric tests. We used Independent Kruskal Wallis test to determine if there were significant differences within our groups at the baseline. Wilcoxon Signed-Rank test was used to determine the groups’ differences from pre-and post-therapy. Consecutively we used Mann-Whitney to determine if there are differences between groups, the analysis was performed by using differential score between the post-test and the pre-test in both groups. The statistical analysis was performed with a 95% confidence level, and significant values were considered to be *p* < 0.05. Descriptive statistics were used to summarize data, including medians and interquartile ranges (IR) for continuous data, for nonparametric analysis. The mean and standard deviation of the parameters were used to calculate the effect size for comparisons using Cohen’s statistics. The size of the effect was classified as small (0.20 to 0.49), medium (0.50 to 0.79) or large (0.8) according to Cohen’s method. We performed the statistical analysis using IBM Statistical Package version 20.0 (SPSS; Chicago, IL, USA).

## 3. Results

Fifty-nine participants completed the study, distributed by computer-generated randomization, out of whom thirty-one subjects in the experimental group, and twenty-eight in the control group. The characteristics of the patients from the investigated groups are presented in Table 2.

No significant differences were found regarding the baseline groups comparison for the outcome measures. As shown in Table 3 and Table 4, results on Related Samples Wilcoxon Signed-Rank test pre- and post-therapy for ROM and MMT, the results suggest significant differences were found in both groups. While for the expermental group ROM for hip internal rotation and plantarflexion were statistically significant pre-and post-therapy, compared to control group, the effect size was small. As it concerns muscle strength, the patients from the experimental group gained muscle force within all planes of motion in ankle movements, compared to the control group, also with a small effect size.

In Table 5 are presented the results of the Wilcoxon Signed-Rank test related to the other types of assessment, and the means for ROM and MMT. The results suggest that VR and MT have influences on the pain scale from FMLE assessment, but also suggests an improvement in the balance of the experimental group compared to the control group.

The Mann-Whitney independent sample test results can be found in Table 6 and provide details on the evolution of ROM and MMT by comparing the control group with the experimental one. The experimental group’s significant differences were registered in hip mobility (flexion, extension, abduction and internal rotation) and plantar flexion, with medium to large effect size. Regarding MMT, at the hip level, the experimental group registered better results than the control group regarding abduction, adduction and external rotation, with a medium effect size. Muscle strength for dorsiflexors and plantar flexors was significantly higher in the experimental group, with a medium to large effect size.

Concerning the comparison between the two groups, from Table 7, the Mann-Whitney test for independent samples results suggests that the experimental group had significantly better outcomes compared to control group regarding ROM, MMT, the motor scale of FMLE and FRT.

## 4. Discussion

Essential features for the nervous system reorganization after a stroke are based on the active implication of cognitive elements, functional specificity and performing complex tasks. By performing complex movements on various patterns and simulating daily activity’s functional movements, the prefrontal cortex, the primary motor cortex, the additional motor area, and the cerebellum are activated [38]. Our study results suggest that combining complex exercises (which converge toward performing various tasks) alongside visual feedback improves cognitive function in controlling the motion. Merging MT with VR therapy, kinesthetic and proprioception training optimizes brain reorganization and LE rehabilitation [39,40]. The results from our sample of patients regarding the ankle movements, both on ROM and muscle strength, suggests that MT, combined with bilateral exercises and foot strengthening exercises, improves muscle strength and ankle stability.

New research on VR as rehabilitation therapy in post-stroke adults highlighted the impact of this type of therapy, with significant improvements in the paretic limb movement rehabilitation, especially in the recovery of balance, the rhythm of executions and gait [41]. It seems that VR is a type of therapy that patients assimilate better than conventional physiotherapy, either because it is new as an approach or because of the diversity of exercises and has an important impact on rehabilitation outcomes [42]. As the outcomes from our research show that both types of interventions have positive and significant results on LE functionality and motor function, it seems that VR therapy associated with MT has better outcomes on ROM, muscle strength and balance. As it was found in previous research, conventional physiotherapy and commercial video-gaming VR exercise improve gait and balance, a rehabilitation VR system, and an adequate protocol can also achieve promising results on LE rehabilitation after stroke [43].

The particular results of ankle strength and balance in the two groups can be approached regarding the foot and ankle’s specific biomechanics and involvement in postural balance. Besides, the study results emphasize the importance of ankle functionality and stability in stroke patients [44]. Another significant contribution on the outcomes is the combination of MT exercises which improves muscle strengths and dedicated VR technology for rehabilitation which incorporates augmented feedback with visual mirror feedback, enriching the individual’s perception of the environment, through visual, hearing and proprioception stimulation [45,46].

The results obtained on hip ROM, for flexion, extension, and abduction, and muscle strength suggests that VR therapy can replace classical physiotherapy in LE rehabilitation, adding more value to the therapeutic exercises program by stimulating the sensorimotor components to enhance brain processes, by combining visual and physical motion stimuli, and visually-inducing self-motion perception [47]. Regarding postural balance training, the VR therapy technology used and the protocol of exercises seem to have better outcomes than standard physical therapy, as the results of FRT suggest. The VR therapy exergames used for postural balance training alter the patient’s perception of the real environment; thus, the patient is more focused on goals achievement than being aware of the danger of falling and being motivated by the cognitive reward succeeding [48].

A major factor in VR rehabilitation is the patients’ ability to understand and execute on command and their mental availability, which plays a key role in the recovery process. Although each exercise is explained before starting, both through the software (visual) and by the therapist (oral), the therapy outcomes are conditioned by the patient’s ability to perform the tasks according to his understanding, which directly depends on the interest and motivation. Nevertheless, one of the reasons VR therapy combined with MT has had better outcomes in LE rehabilitation in the chronic post-stroke phase is the real positive influence of VR on patients’ motivation and cognitive outcomes [49].

Our research aimed to identify if a protocol of exercises for lower limb VR training after stroke, combined with MT exercises, has better outcomes than standard physiotherapy. The results suggest increasing rehabilitation outcomes by integrating VR and MT in the rehabilitation program by stimulating LE motor and functional rehabilitation and postural balance.

Although in the last decade, the emphasis has been focused on rehabilitation therapy within the first 3–6 months post-stroke, lately, it has been shown that neuroplasticity may occur in chronic post-stroke stages. If the patient is included in rehabilitation programs, the restoration and the maintenance of motor functions and paretic limbs functionality may occur [50,51]. Patient rehabilitation is essential both at the individual and family level and at the level of society and the health system. It is well known that both the individual and the family are actively included in the post-stroke rehabilitation process alongside the multidisciplinary team (rehabilitation physician, nurses, physiotherapists, speech therapist, psychologists, occupational therapists, orthosis specialists) [52]. Even more, any case of a post-stroke patient with significant motor disabilities, impossible to perform ADLs, cannot fulfill family and professional tasks, is a tremendous burden individually, socially and for the healthcare system [53]. And like most disabling conditions, it involves depression and needs multidisciplinary resources for the physical and mental rehabilitation of the individual and their caregivers [54]. Additionally, the relationship between the patient and the physiotherapist, the level of communication between the two, and the patient’s ability to understand and comply with the needed tasks contribute to the process of physical rehabilitation, often neglected aspects in the Romanian medical system [55]. Thus, through the use of VR, MT, and other new methods of training and rehabilitation of patients in the chronic post-stroke stages, it is possible to interfere in the health status, improving the individual’s functional capacity, with chain repercussions on the family and society.

Our research’s primary limitation is the short length of patient rehabilitation (two weeks). A secondary one is a lack of follow up at several intervals of time after the therapy ended. We must underline that the short period of treatment is imposed by the national health system’s policy and the Health Assurance System in Romania, and thus, the duration of hospitalization could not be increased. Furthermore, we want to emphasize the need for cooperation between the social and healthcare systems, especially for patients with disabilities, to assure the continuity of the rehabilitation program and nursing care. The need to advocate for government policies, both national and European or why not at the international level, becomes obvious to reduce the burden borne by the individual, caregivers, society, and healthcare system and increase each patient’s life quality. We must also discuss the fact that no standardized tools have been used to determine the parameters of gait and balance, except for FRT and TUG assessments, revealing the need for future research to assess the results obtained systematically.

## 5. Conclusions

The results of our research suggest that combining VR rehabilitation technology with MT exercises creates a more favorable environment for lower extremity rehabilitation in chronic patients after stroke, by combining several types of feedback, with an increased impact on neuroplasticity. Our study results are promising compared to standard physiotherapy, and the combination of several types of interventions on the chronic post-stroke patient must be further studied to find the best solutions to reduce disability and burden borne by the individual family, society, and health systems.

## Figures and Tables

**Table 1:** Types of physiotherapy interventions.

Types of Interventions	Experimental Group	Control Group	Minutes of Therapy
Passive analytical exercises for each LE joint	No	Yes	10
Prone or supine active analytical exercises for LE joint	No	Yes	20
Active LE exercises from standing position, and proprioception	No	Yes	20
Active ergometer bicycle	Yes	Yes	10
Treadmill	Yes	Yes	10
VR exercises	Yes	No	32 ± 5
MT exercises for ankle	Yes	No	18 ± 5
Total time of physiotherapy exercises (minutes)	70	70	70

LE—Lower Extremity, VR—Virtual Therapy, MT—Mirror Therapy.

**Table 2:** Patient characteristics.

Characteristic	Experimental (*n* = 31)	Control (*n* = 28)	*p*
Affected side (left/right)	16/15	8/20	0.074
Hemorrhagic/Ischemic stroke	18/13	13/15	0.821
Gender (male/female)	7/24	8/20	0.601
Age (mean/SD)	59.03/10.12	60.67/8.17	0.767
Post-stroke Time (mean/SD)	2.74/1.10	2.71/0.99	0.456
Mean (minutes)/SD VR Duration	22.16/4.01	0	0.317
Total Physiotherapy Duration (minutes)	70	70	1.00

**Table 3:** Related Samples Wilcoxon Signed-Rank test pre-and post-therapy for ROM and MMT, for experimental group.

	ROM				MMT			
	Pre-Therapy	Post-Therapy			Pre-Therapy	Post-Therapy		
	Median (IR)	Median (IR)	*p*	Cohen’s d	Median (IR)	Median (IR)	*p*	Cohen’s d
Hip Flexion	85.00 (5.00)	90.00 (10.00)	0.000	0.601	3.50 (0.75)	3.75 (0.75)	0.000	0.538
Hip Extension	10.00 (5.00)	10.00 (5.00)	0.000	0.833	3.25 (0.50)	3.50 (0.75)	0.000	0.834
Hip Abduction	30.00 (15.00)	35.00 (10.00)	0.000	0.853	3.25 (0.75)	3.50 (0.75)	0.000	0.718
Hip Adduction	0/0	0	0.083	0.090	3.25 (0.50)	3.50 (0.75)	0.000	0.574
Hip Internal Rotation	10.00 (10.00)	10.00 (15.00)	0.002	0.317	2.00 (1.75)	2.50 (2.00)	0.001	0.204
Hip External Rotation	25.00 (15.00)	25.00 (20.00)	0.000	0.349	2.75 (0.75)	3.00 (0.75)	0.011	0.256
Knee Flexion	80.00 (15.00)	90.00 (15.00)	0.000	0.443	3.25 (1.00)	3.25 (0.50)	0.000	0.573
Knee extension	0	0	1.000	NA	3.50 (0.75)	3.75 (0.50)	0.000	0.536
Dorsiflexion	15.00 (10.00)	20.00 (12.00)	0.000	0.468	2.75 (1.25)	3.00 (1.25)	0.000	0.302
Plantarflexion	10.00 (15.00)	15.00 (17.00)	0.000	0.436	3.00 (2.00)	3.00 (1.25)	0.000	0.295
Inversion	10.00 (24.00)	10.00 (25.00)	0.002	0.182	2.00 (1.75)	2.00 (2.00)	0.007	0.134
Eversion	6.00 (20.00)	10.00 (25.00)	0.001	0.209	1.75 (2.00)	2.00 (1.75)	0.002	0.238

ROM—Range of Motion, MMT—Manual Muscle Testing, IR—Interquartile Range, NA—Not Available.

**Table 4:** Related Samples Wilcoxon Signed-Rank test pre-and post-therapy for ROM and MMT, for control group.

	ROM				MMT			
	Pre-Therapy	Post-Therapy			Pre-Therapy	Post-Therapy		
	Median (IR)	Median (IR)	*p*	Cohen’s d	Median (IR)	Median (IR)	*p*	Cohen’s d
Hip Flexion	83.00 (27.50)	90.00 (13.75)	0.011	0.324	3.25 (0.50)	3.50 (0.50)	0.000	0.521
Hip Extension	5.00 (5.00)	10.00 (5.00)	0.010	0.419	3.25 (0.44)	3.50 (0.75)	0.001	0.456
Hip Abduction	22.50 (10.00)	27.50 (9.25)	0.045	0.382	3.12 (0.50)	3.25 (0.75)	0.001	0.458
Hip Adduction	0.00 (0.00)	0.00 (0.00)	1.000	NA	3.00 (0.44)	3.25 (0.75)	0.006	0.436
Hip Internal Rotation	10.00 (13.75)	12.50 (12.25)	0.052	0.250	2.75 (1.19)	2.87 (0.75)	0.001	0.354
Hip External Rotation	15.00 (16.25)	20.00 (13.75)	0.006	0.144	2.50 (0.94)	2.50 (0.94)	0.025	0.064
Knee Flexion	75.00 (35.00)	80.00 (25.00)	0.000	0.337	3.00 (0.25)	3.50 (0.44)	0.000	0.814
Knee extension	0	0	1.000	NA	3.25 (0.50)	3.50 (0.69)	0.000	0.681
Dorsiflexion	15.00 (9.50)	15.00 (25.00)	0.005	0.449	3.00 (1.13)	3.00 (1.19)	0.133	0.111
Plantarflexion	20.00 (15.00)	20.00 (13.75)	0.630	0.057	3.00 (1.31)	3.00 (1.63)	0.283	0.131
Inversion	15.00 (10.00)	20.00 (25.00)	0.002	0.325	2.12 (1.44)	2.12 (1.48)	0.059	0.050
Eversion	6.00 (10.00)	10.00 (18.25)	0.000	0.451	2.12 (1.75)	2.12 (1.80)	0.265	0.018

ROM—Range of Motion, MMT—Manual Muscle Testing, IR—Interquartile Range, NA—Not Available.

**Table 5:** Related Samples Wilcoxon Signed-Rank test pre-and post-therapy.

	Experimental Group				Control Group			
	Pre-Therapy	Post-Therapy			Pre-Therapy	Post-Therapy		
	Median (IR)	Median (IR)	*p*	Cohen’s d	Median (IR)	Median (IR)	*p*	Cohen’s d
ROM (mean)	39.17 (5.42)	42.92 (7.42)	<0.001	0.645	37.71 (7.40)	38.83 (8.56)	<0.001	0.504
MMT (mean)	2.89 (1.02)	3.05 (0.81)	<0.001	0.533	2.86 (0.73)	3.07 (0.81)	<0.001	0.324
MAS	2.00 (2.00)	2.00 (2.00)	0.157	−0.049	1.00 (1.00)	1.00 (1.00)	1.000	0.00
MRS	2.00 (1.00)	2.00 (1.00)	1.000	0.00	2.00 (0.75)	2.00 (0.75)	1.000	0
Motor FMLE	24.00 (6.00)	27.00 (7.00)	0.000	0.685	24.50(6.75)	25.5 (6.50)	0.000	0.254
Passive FMLE	17.00 (5.00)	20.00 (2.00)	0.136	0.466	17.50 (3.75)	18.00 (3.75)	0.034	0.121
Pain FMLE	20.00 (0.00)	20.00 (0.00)	0.020	0.212	20.00 (4.50)	20.00 (3.00)	0.063	0.157
FIM	114.00 (13.00)	114.00 (13.00)	1.000	0	121.00 (15.75)	121.00 (15.75)	0.157	0.010
TUG	2.00 (0.00)	2.00 (1.00)	0.083	0.011	2.00 (0.00)	2.00 (0.00)	0.317	0.075
FRT	25.00 (8.00)	27.00 (5.30)	0.000	0.573	23.00 (11.00)	23.00 (12.75)	0.470	0.004

IR—Interquartile, Range ROM—Range of Motion, MMT—Manual Muscle Testing, FMLE: Fugl-Meyer Assessment for Lower Extremity; MRS: Modified Rankin Scale, FIM: Functional Independence Measure; MAS: Modified Ashworth Scale; TUG—Timed Up and Go test FRT: Functional Reach Test.

**Table 6:** Mann-Whitney test for independent samples for ROM and MMT.

	ROM				MMT			
	Experimental Group	Control Group			Experimental Group	Control Group		
	Median (IR)	Median (IR)	*p*	Cohen’s d	Median (IR)	Median (IR)	*p*	Cohen’s d
Hip Flexion	5.00 (9.00)	0.00 (5.00)	0.001	0.611	0.25 (0.25)	0.25 (0.19)	0.199	0.117
Hip Extension	5.00 (5.00)	0.00 (2.00)	0.000	0.929	0.25 (0.25)	0.25 (0.50)	0.140	0.261
Hip Abduction	5.00 (10.00)	0.00 (4.50)	0.006	0.518	0.25 (0.50)	0.00 (0.25)	0.030	0.568
Hip Adduction	0.00 (0.00)	0.00 (0.00)	0.094	NA	0.25 (0.50)	0.00 (0.75)	0.012	0.610
Hip Internal Rotation	0.00 (5.00)	0.00 (2.00)	0.593	0.159	0.00 (0.25)	0.00 (0.25)	0.276	0.031
Hip External Rotation	3.00 (10.00)	0.00 (5.00)	0.028	0.761	0.00 (0.50)	0.00 (0.00)	0.011	0.792
Knee Flexion	5.00 (13.00)	5.00 (10.00)	0.249	0.287	0.25 (0.50)	0.25 (0.25)	0.146	0.290
Knee extension	0.00 (0.00)	0.00 (0.00)	1.000	NA	0.25 (0.50)	0.25 (0.75)	0.828	0.134
Dorsiflexion	3.00 (8.00)	0.00 (8.75)	0.386	0.244	0.25 (0.75)	0.00 (0.25)	0.001	0.870
Plantarflexion	5.00 (5.00)	0.00 (2.00)	0.003	0.752	0.25 (0.50)	0.00 (0.00)	0.002	0.520
Inversion	0.00 (5.00)	0.00 (5.00)	0.354	0.299	0.00 (0.25)	0.00 (0.00)	0.151	0.439
Eversion	0.00 (5.00)	2.00 (5.00)	0.383	0.346	0.00 (0.25)	0.00 (0.25)	0.223	0.545

**Table 7:** Mann-Whitney test for independent samples.

	Experimental Group		Control Group			
	Median (IR)	Mean Rank	Median (IR)	Mean Rank	*p*	Cohen’s d
ROM	3.75 (3.25)	36.65	2.08 (2.27)	22.64	0.002	0.693
MMT	0.27 (0.35)	36.34	0.10 (0.15)	22.98	0.003	0.924
MAS	0.00 (0.00)	29.10	0/0	31.00	0.175	0.365
MRS	0.00 (0.00)	30.00	0/0	30.00	1.000	NA
Motor FMLE	3.00 (2.00)	38.48	1.00 (2.00)	20.61	0.000	0.984
Passive FMLE	0.00 (1.00)	34.69	0.00 (0.00)	33.80	0.008	0.727
Pain FMLE	0/0	30.42	0.00 (0.00)	29.54	0.763	0.155
FIM	0/0	29.00	0.00 (0.00)	31.11	0.133	0.385
TUG	0/0	30.85	0.00 (0.00)	29.05	0.356	0.243
FRT	2 (2.50)	37.15	0.00 (1.75)	22.09	0.001	0.936

FMLE: Fugl-Meyer Assessment for Lower Extremity; MRS: Modified Rankin Scale, FIM: Functional Independence Measure; ROM: Range of Motion; MMT: Manual Muscle Testing; MAS: Modified Ashworth Scale; TUG-Timed Up and Go t FRT: Functional Reach Test.

## Data Availability

Data of this paper can be accessed through a standard application procedure according to local health data-sharing regulation.

## Supplementary Materials

The following are available online at https://www.mdpi.com/1660-4601/18/5/2654/s1, Supplementary file: Lower extremity rehabilitation in patients with post-stroke sequelae through virtual reality associated with mirror therapy—supplementary file with Mirror Therapy and Virtual Reality exergames examples.
