# Supplementary material for: Lower Extremity Rehabilitation in Patients with Post-Stroke Sequelae through Virtual Reality Associated with Mirror Therapy
Source: Int J Environ Res Public Health. 2021 Mar 6;18(5):2654. doi: 10.3390/ijerph18052654 (PMC7967355; doi:10.3390/ijerph18052654)

Lower extremity rehabilitation in patients with post-stroke sequelae through virtual reality associated with mirror therapy- supplementary file with Mirror Therapy and Virtual Reality exergames examples

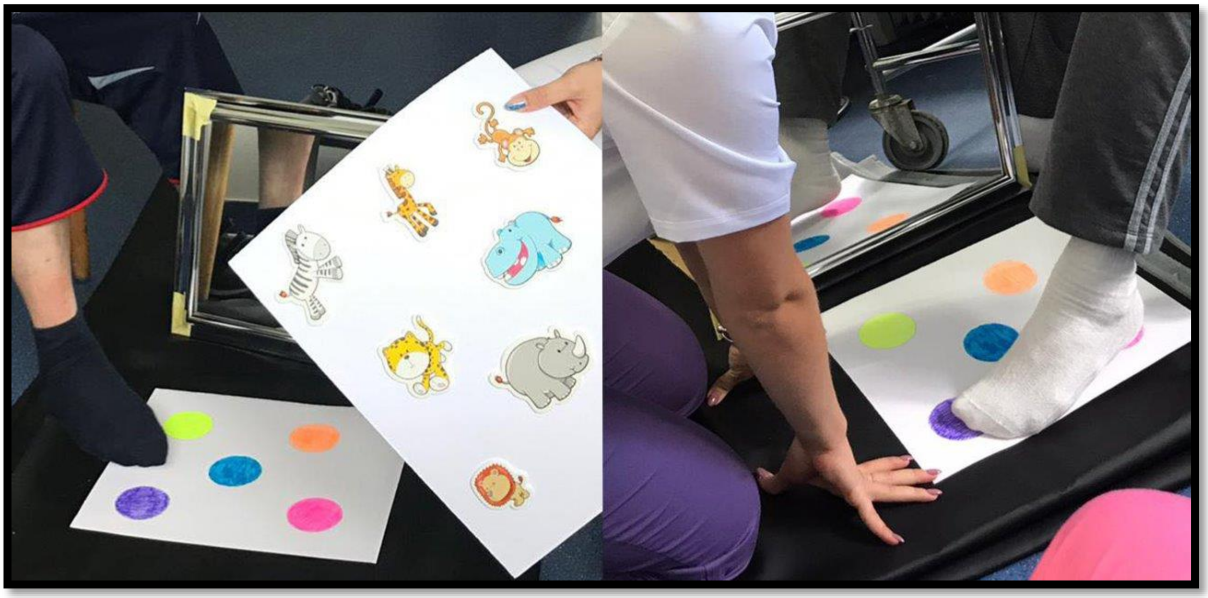

Exercise performed by Mirror Therapy

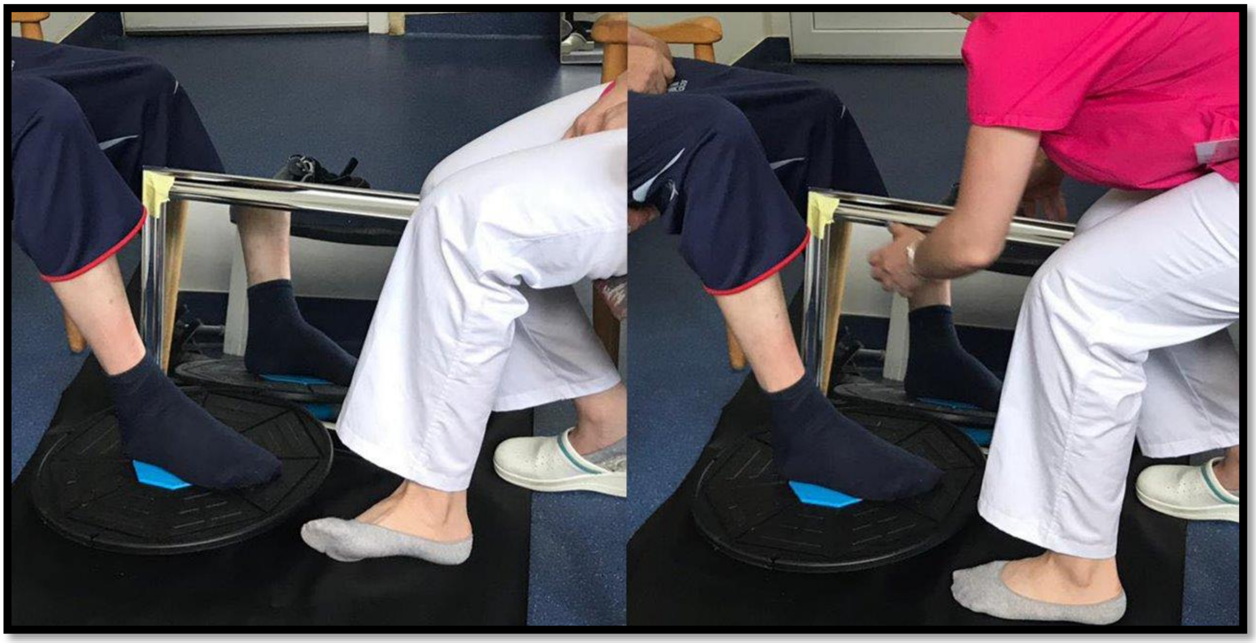

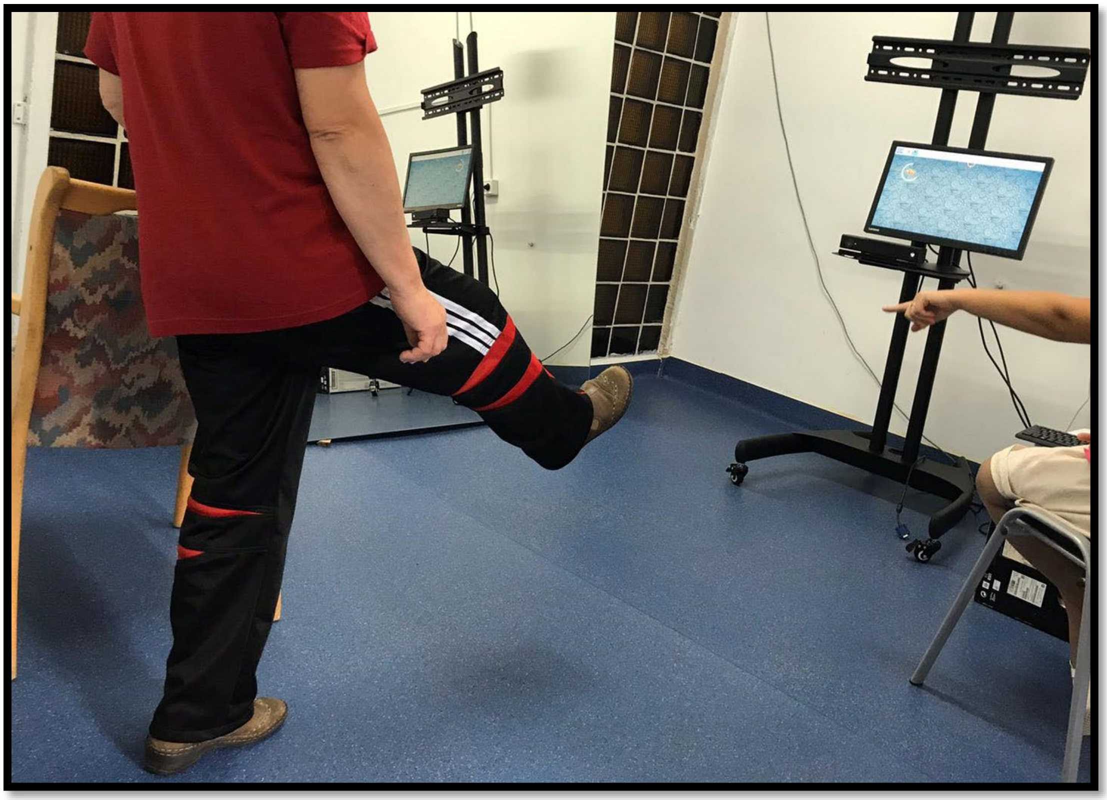

Exergame- Catch, where the patient shall move his LE randomly (left, right, up down) to „catch” with his foot the elements on screen.

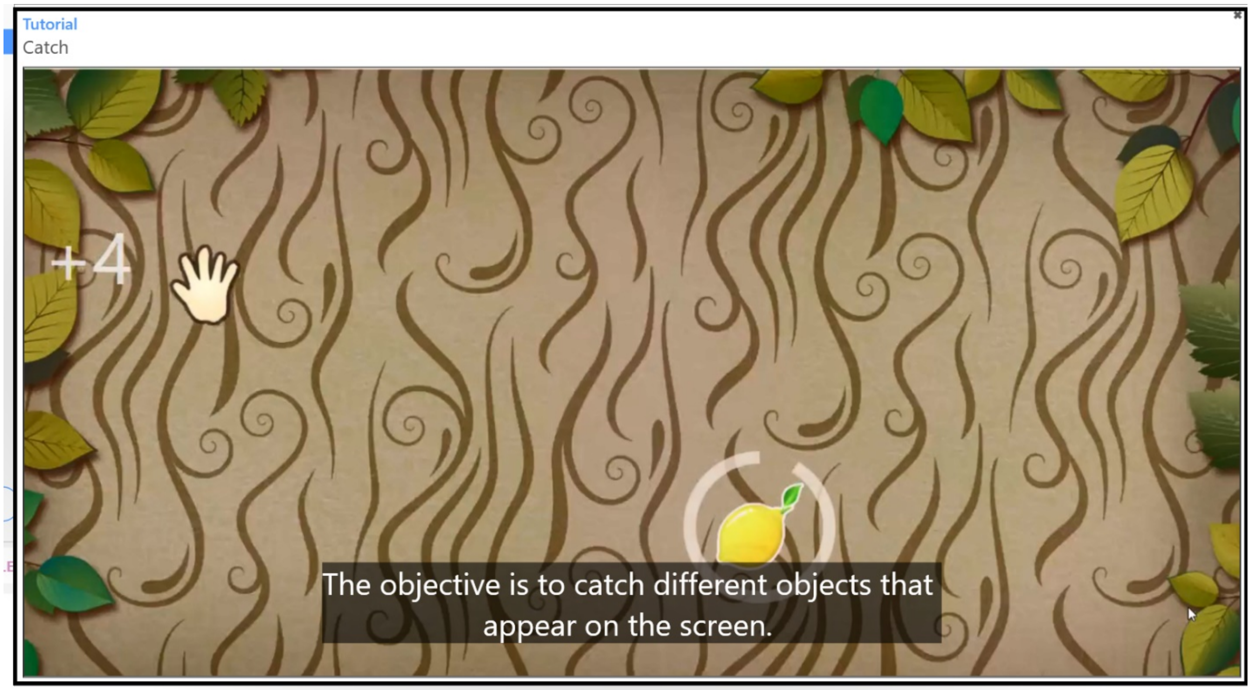

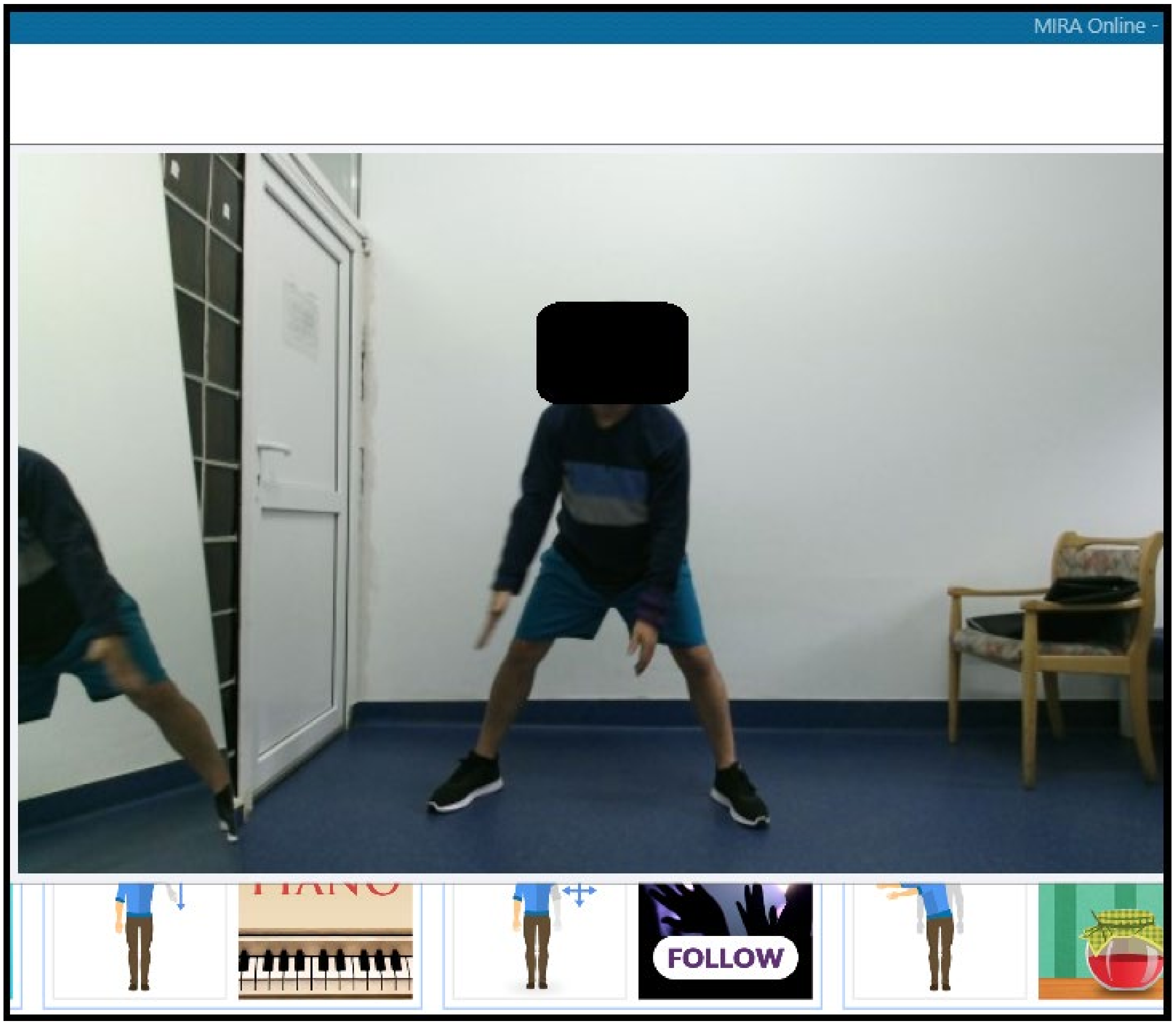

Exergame „Grab", involves catching objects on shelves, with both hands and placing them on various levels and locations, without the patient detaching his feet from the floor.

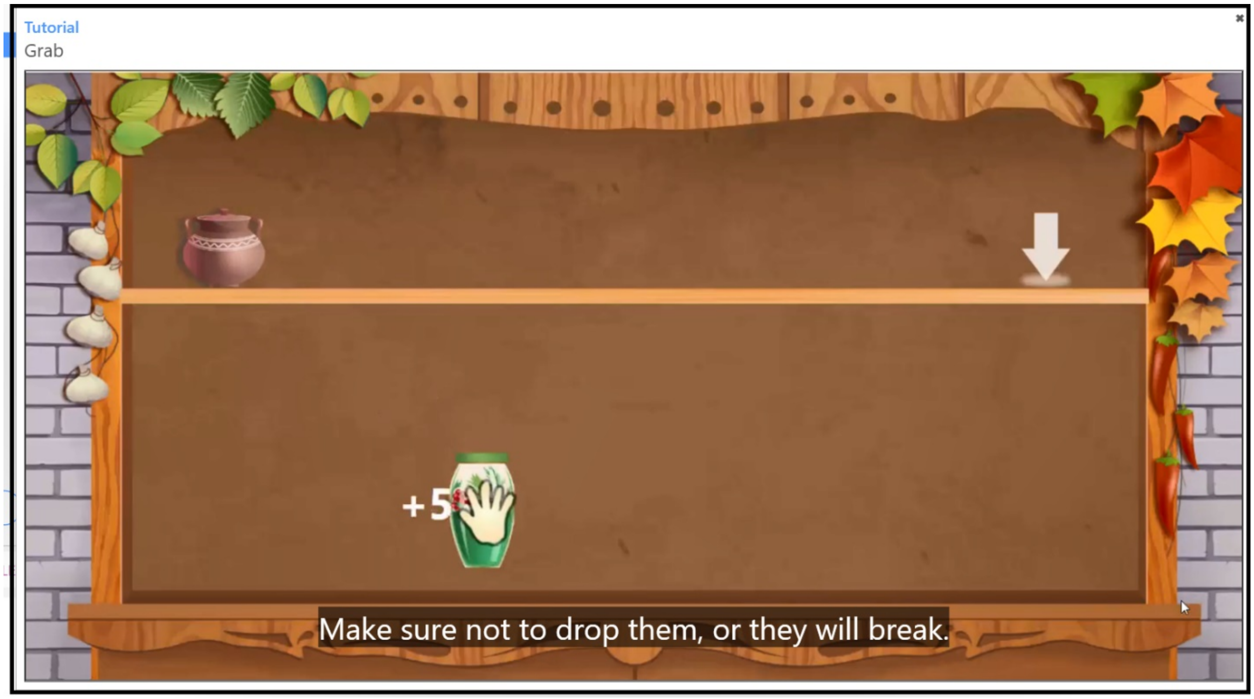

Supplement: Supplementary file 1 [file ijerph-18-02654-s001.pdf]
